# The Rising Global Cesarean Section Rates and Their Impact on Maternal and Child Health: A Scoping Review

**DOI:** 10.3390/jcm14228102

**Published:** 2025-11-15

**Authors:** Sofia Thomaidi, Antigoni Sarantaki, Maria Tzitiridou Chatzopoulou, Eirini Orovou, Vaidas Jotautis, Dimitrios Papoutsis

**Affiliations:** 1 Midwifery Department, University of Western Macedonia, 50200 Ptolemaida, Greece; dmw00031@uowm.gr (S.T.); dpapoutsis@uowm.gr (D.P.); 2 Midwifery Department, Faculty of Health and Care Sciences, University of West Attica, Egaleo, 12243 Athens, Greece; 3 Faculty of Medicine, Kauno Kolegija Higher Education Institution, Pramonės pr 20, 50468 Kaunas, Lithuania; vaidas.jotautis@go.kauko.lt

**Keywords:** cesarean section, short-term effects, long-term effects, mother’s health, child’s health, health system

## Abstract

**Background**: A cesarean section (CS) is a method of childbirth involving a surgical cut made in the abdominal and uterine wall to deliver the infant. But while it saves the lives of women and infants, it has been implicated in several immediate and long-term complications and adverse consequences as a result of its ineffective use. This study attempts to address the major public health issue of the inappropriate use of CS by exploring its impact on maternal, neonatal, and child health. More specifically, the study aims to investigate the immediate and long-term health impacts on the mother, including her physical and mental health, as well as the immediate and long-term psychosomatic consequences on the neonate’s, infant’s, and child’s health as a result of the inappropriate use of CS. **Methods**: A scoping review was conducted spanning from 2015 to 2025 according to the PRISMA criteria and checklist. Searches were performed in PubMed, Scopus, and Google Scholar. The inclusion criteria comprised original articles with clear exposure and outcome written in English, and studies that did not involve reviews of any kind or letters to the editors. **Results**: The review identified 42 relevant studies. The results showed several immediate and long-term complications of CS in mothers, neonates, infants, and children, while its ineffective use carries equally high risks, such as high levels of morbidity and mortality. **Conclusions**: This scoping review presents the problems that arise in the immediate and long-term health of mother and child from the improper use of cesarean section and underlines the need for immediate action and measures to be taken by health policy makers.

## 1. Introduction

Cesarean section (CS) is a surgical procedure used to deliver the baby through an incision in the mother’s abdomen. It is typically recommended when a vaginal delivery (VD) may pose risks to the health of the mother, baby, or both [1]. While CS is a life-saving procedure in cases of complications, its excessive use without medical indications jeopardizes the physical and mental well-being of women and children [1]. Over the past few decades, there has been a significant global rise in the rate of CS, from around 7% in 1990 to 21% today, and this has emerged as a pressing public health concern [2]. It seems that these trends are expected to continue their upward trajectory during the current decade, with unmet needs and overuse coexisting, leading to a global rate that is projected to reach 29% by 2030 [1]. However, the World Health Organization (WHO) recommends that CS rates should ideally range between 10 and 15%, as rates beyond this threshold do not correlate with better maternal or neonatal outcomes [1]. According to this WHO statement, the underuse of CSs may lead to an increase in perinatal maternal and neonatal morbidity and mortality. On the other hand, their use without medical indication has not shown any benefit and, moreover, can significantly contribute to the waste of human and material resources. At this point, it should be noted that due to health inequalities, some countries face unsafe access to CSs, while others overuse them with adverse effects on maternal and neonatal health. Based on the above, the inappropriate use of CSs is a matter of global concern and a significant challenge for public health.

Regarding CS trends, in 2018, the global CS rate was 21.1%, with an average of 8.2% in developing countries, 24.2% in less developed countries, and 27.2% in developed countries [3]. At the two extremes of CS rates are Sub-Saharan African countries with the lowest rate (5%) and the countries of Latin America and the Caribbean with the highest rate (42.8%). The five countries with the highest cesarean section (CS) rates globally were the Dominican Republic (58.1%), Brazil (55.7%), Cyprus (55.3%), Egypt (51.8%), and Turkey (50.8%), which also recorded the highest rates in the Americas, Asia, and Africa. In Europe, Romania had the highest CS rate at 46.9%. On the other hand, the lowest CS rates in the world were observed in Africa, with Chad (1.4%), Niger (1.4%), Ethiopia (1.9%), Madagascar (2%), and Cameroon (2.4%). In other regions, the lowest rates were recorded in Timor-Leste (3.5%) in Asia, Papua New Guinea (3.0%) in Oceania, the Netherlands (14.9%) in Europe, and Haiti (5.4%) in Latin America [3]. Regarding Greece, official CS rates have not been recorded; however, research indicates very high rates, reaching up to 58% [4]. The continuous increase in CS rates suggests that by 2030, global rates may reach 30%. Sub-Saharan Africa is expected to remain with rates lower than 10%, while in regions such as Eastern and Western Asia and Latin America, CS may become the dominant method of delivery [3].

On the other hand, CSs are not solely conducted for medical reasons, as various other factors can influence the decision to choose this procedure. These reasons include the mother’s desire [5] due to perceived anxiety or fear of vaginal birth pain [6], the preference for a scheduled delivery on a specific date [7], the convenience for the doctor, and financial incentives for doctors or hospitals, which lead to higher CS rates in private hospitals compared to public ones. Additionally, various religious reasons and social beliefs may influence a woman’s decision to deliver via CS [8]. Moreover, the fear of legal repercussions and the potential for litigation, as secondary negative consequences of vaginal delivery, have been identified as significant factors influencing clinicians’ decisions to opt for CS as a precautionary measure, contributing to the increase in CS deliveries [9]. In recent years, ambiguity has emerged regarding what healthcare professionals consider to be clinical indications for CS. Changing risk profiles and maternal characteristics, such as increased maternal age [5], high body mass index (BMI) [10], and infertility treatments [11], are reported to contribute to the rise in CS rates. An additional crucial element influencing CS rates is the presence of midwives. Greater accessibility to midwifery services is linked to CS rates, aligning more closely with the recommended range of 10–15% [12]. Taking into account the international trends of cesarean sections, the recommendations of the WHO, and the consequences of inappropriate or improper use of cesarean sections, it is appropriate to further investigate the issue.

Recent global evidence underscores that while CS is a life-saving procedure, it is also associated with a broad spectrum of short- and long-term complications for both mothers and children. Maternal complications include increased risks of hemorrhage, infection, abnormal placentation (such as placenta previa and accreta), thromboembolic events, chronic pelvic pain, and infertility in subsequent pregnancies [13]. Neonatal and child outcomes related to CS have also been well documented, including respiratory morbidity, alterations in gut microbiota, immune and metabolic disorders, and increased susceptibility to obesity and allergies during childhood [14,15]. Moreover, women undergoing CS report higher rates of postpartum depression and post-traumatic stress symptoms compared to those experiencing vaginal birth. The overall burden of CS-related morbidity contributes substantially to healthcare costs and long-term public health challenges, emphasizing the need for cautious and evidence-based use of this surgical procedure [16,17].

However, a particular long-term complication of CS that has gained increasing attention in recent years is isthmocele, also known as a cesarean scar defect or uterine niche. Isthmocele is a dehiscence or pouch-like defect at the site of the uterine scar, often identified through imaging [18]. It may remain asymptomatic or cause abnormal uterine bleeding, dysmenorrhea, chronic pelvic pain, infertility, or secondary infertility due to altered uterine contractility and sperm transport [18,19]. Additionally, isthmocele increases the risk of abnormal placentation, cesarean scar ectopic pregnancy, and uterine rupture in subsequent pregnancies. As global CS rates continue to rise, the clinical recognition and management of isthmocele have become an emerging aspect of reproductive health and obstetric care, highlighting the long-term reproductive implications of surgical delivery [20].

This study attempts to address this major public health issue by exploring the impact of CS on maternal, neonatal, and child health. More specifically, the study aims to investigate the immediate and long-term health impacts on the mother, including her physical and mental health, as well as the immediate and long-term psychosomatic consequences on the neonate’s, infant’s, and child’s health as a result of the Inappropriate Use of CS.

## 2. Materials and Methods

This scoping review was conducted in accordance with the PRISMA-ScR (Preferred Reporting Items for Systematic Reviews and Meta-Analyses extension for Scoping Reviews) [21] guidelines to identify the short-term and long-term complications of CS in mothers and newborns, infants, and children, as well as the complications arising from its ineffective use, based on the existing literature. The review protocol was not registered. It was conducted using the methodological steps outlined by Mak and Thomas (2022): (a) the identification of the research question; (b) the identification of the relevant studies; (c) selection of appropriate studies to be included in the review; (d) charting the data; and (e) collating, summarizing, and reporting the results [22]. The PRISMA-ScR checklist is provided in the Supplementary Materials.

### 2.1. Eligibility Criteria

The process of selecting relevant studies began with the establishment of clear inclusion and exclusion criteria. Quantitative studies published in English from 2010 to the present, which had CS as the exposure and mother–child outcomes as the effect, were included. Therefore, studies that did not have clear exposure and outcome variables, studies written in a language other than English, and studies that involved reviews of any kind or letters to the editors were excluded.

### 2.2. Search Strategy

**Figure 1:**
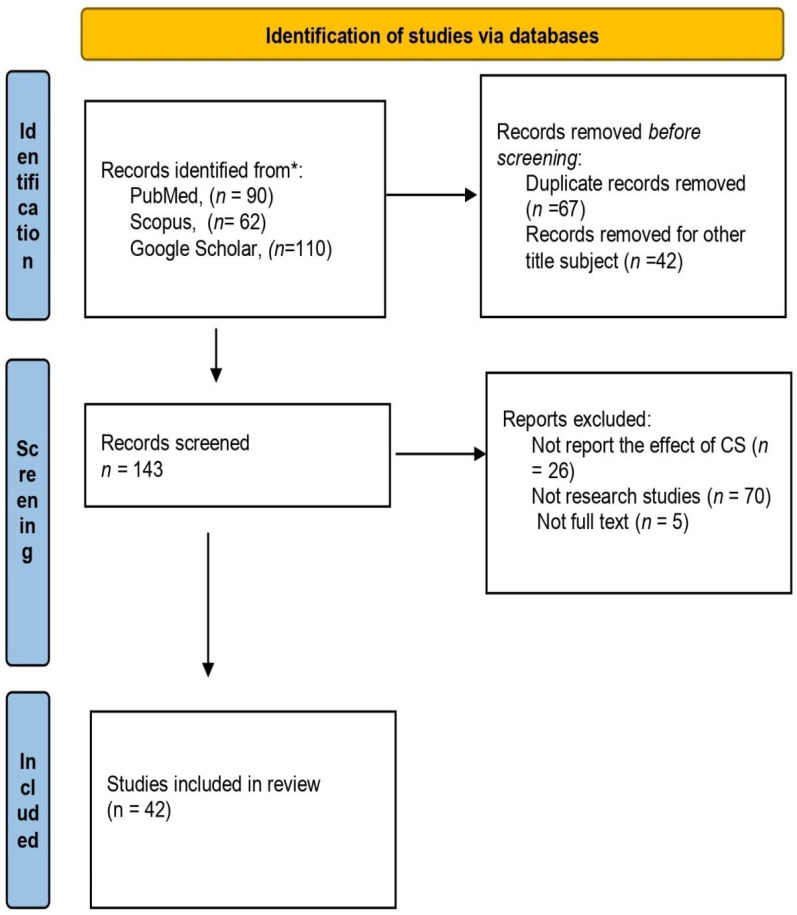
Flow chart—structure search strategy. * The databases are listed in detail to clarify the search process.

We searched published papers with the following databases: PubMed/Medline, Crossref, and Google Scholar, from December 2024 to January 2025. The search terms used were as follows: ((((cesarean section) OR (cesarean delivery)) AND (maternal outcomes)) OR (maternal mortality)) OR (maternal morbidity) OR (inappropriate use) OR, (((cesarean section) OR (cesarean delivery)) AND (neonatal outcomes)) OR (infant outcomes) OR (child outcomes))). The selection process was carried out in accordance with the recommended PRISMA methodology (Figure 1). The initial search yielded a total of 1913 records the following three databases: PubMed (*n*  =  90), Scopus (*n*  =  62), and Google Scholar (*n*  =  110). We removed 67 duplicate papers before screening. Furthermore, we excluded 42 records due to their irrelevance based on their title and subject matter. Also, we removed 26 papers which were not reported in the outcome factor of our study, 70 studies which were not original papers, as well as 5, and another 5 that were not written in English. Finally, 42 original articles were included in our review.

## 3. Results

### 3.1. Short-Term and Long-Term Health Effects of CSs on Mothers

Understanding the immediate and long- and short-term effects of CS on women is limited by issues in study design, insufficient statistical power, lack of control for confounding factors, and inappropriate selection of comparison groups. Furthermore, a clear distinction has not been made between elective and emergency CSs or the obstetric and medical parameters leading to the need for CS delivery, which may be associated with increased morbidity and mortality. A review of data on the short and long-term effects on women is provided in Table 1, including increased risks of hysterectomy [23], abnormal placentation [24], uterine rupture [23], stillbirth [25], and preterm birth in future pregnancies [26,27], the need for blood transfusions [23,28], pelvic adhesions [29], intraoperative injuries [23], hysterectomy and cesarean scar ectopic pregnancies [26], which are more common as the number of CS increases. Emergency CSs are associated with a higher incidence of immediate maternal complications compared to elective ones [30], such as infection, fever, urinary tract infections [31], wound dehiscence [32], disseminated intravascular coagulation, and reoperation; this reflects the extent of prenatal complications and the need for immediate intervention [30]. However, a history of risk of maternal mortality during subsequent delivery due to the presence of abdominal adhesions and hemorrhage [33]. Maternal mortality was observed when CS rates were below the percentage recommended by the WHO (5–10%) [34]. On the contrary, CS rates exceeding the WHO threshold are not associated with reduced mortality outcomes [35]. However, direct maternal mortality linked to elective CSs, without adjusting for pre-labor risk factors, varied between 0 and 47 deaths per 100,000 procedures. The relative risk of death with elective CS compared to vaginal delivery was estimated at 0.45 [36].

On the other hand, CS can also affect maternal mental health and well-being, reducing women’s postpartum adjustment and contributing to an increased risk of developing post-traumatic and depressive symptoms [16]. It appears that the frustration of birth expectations [37], the sight of the surgical room [38], increased pain [39], and difficulties with breastfeeding [40], reduce psychological resilience, combined with other factors, such as a psychiatric history and insufficient support from the significant others [37], the likelihood of birth trauma and, consequently, mental illness during the postpartum period increases.

**Table 1:** Short- and long-term effects of cesarean section on mothers.

Author/Year/ Country	Women’s Sample	Design	Acute Maternal Outcomes	Chronic Maternal Outcomes	Maternal Well-Being
Lannon et al., 2015 [41], USA	10,505	Cohort study	-	Uterine rupture in the subsequent pregnancy	-
Bowman et al., 2015 [26], USA	260,249	Cohort study	-	Increased risk of cesarean scar ectopic pregnancies	-
Aslı Sis Çelik, [42], 2015, Turkey	131	Comparative and descriptive study	-	-	Difficulty recovering and more pain
Butwick et al., 2017 [33], USA	819	Case–control study	Hemorrhage	-	-
Matalliotakis et al., 2017 [43], Greece	76	Prospective study	-	Placenta previa	-
Gundersen et al., 2018 [31], Denmark	45,053	Retrospective study	Urinary tract infections	-	-
Kjerulff & Brubaker, 2018 [44], USA	3006	Cohort study	-	-	Feelings of frustration
Guglielminotti et al., 2019 [45], USA	466,014	Retrospective study	Anesthetic-related complications	-	-
Alshehri et al., 2019 [29], Saudi Arabia	394	Case–control study	-	Ιntraoperative bleeding due to adhesions and complete or incomplete rupture of scar	-
Schobinger et al., 2020 [38], Switzerland	647	Prospective study	-	-	Unfulfilled expectations and psychological trauma
Orovou et al., 2020 [37], Greece	166	Prospective study	-	-	Unfulfilled expectations and psychological trauma
Kjerulff et al., 2020 [25], USA	712	Cohort study	-	Lower conception rate, stillbirth or miscarriages, and sub-fertility	-
Kayem et al., 2020 [24], France	396	Retrospective study	-	Placenta previa and placenta accreta	-
Carbonnel et al., 2021 [32], France	1520	Retrospective study	Wound complications	-	-
Hou et al., 2022 [46], China	358	Cross-sectional study	-	-	Tokophobia
Wei et al., 2022 [47], China	1411	Retrospective study	-	Chronic endometritis	-
Larsson et al., 2022 [28], Sweden	614,355	Retrospective study	Cardiac arrest, intubation, and inclusion in ICU	-	-
J. Singh et al., 2022 [40], Canada	418	Prospective study	-	-	Difficulties in breastfeeding
Odada et al., 2024 [48], Nairobi	1262	Retrospective study	Surgical site infections	-	-
de Vries et al., 2024 [23], France	5422	Retrospective study	Maternal death due to uterine atony, severe hemorrhage, and surgical injury during CS	-	-
Kathpalia et al., [49], 2024, India	10,296	Retrospective study	-	Abnormal placentation	-
Woolner et al., 2024 [27], UK	30,253	Cohort study	-	Preterm births, especially if the first CS was performed at full dilation	-

Notes: A cute maternal outcomes occur immediately after the CS until the end of the postpartum period. Chronic outcomes may occur immediately after the postpartum period or after months or years [50]. Studies include retrospective, prospective, and case–control designs. Most data originate from high-income countries (USA, France, and UK), with few contributions from middle-income regions (e.g., Greece, Turkey, and Saudi Arabia).

### 3.2. Short-Term and Long-Term Health Effects of CSs on Neonates, Infants, and Children

Cesarean sections undoubtedly play undeniably save the neonate’s lives and reduce perinatal mortality and severe complications like intrauterine asphyxia. However, scheduling elective CS before 39 weeks of gestation may elevate the risk of respiratory problems and hypoglycemia in neonates [51]. As a result, the risk of NICU admission and respiratory complications is common in neonates following CS, while the long-term effects include frequent respiratory infections and asthma in childhood [51]. Neonates delivered via CS before the onset of spontaneous labor are more likely to be admitted to the NICU [52].

It is well established that interventions during childbirth and the mode of delivery can have both immediate and long-term effects on neonatal health. This impact is thought to be linked to the insufficient transfer of maternal microbiota (gut and skin) to the neonate during CS, a phenomenon whose implications for later life are still under investigation [53]. Abnormal gut colonization may influence the neonate’s immune system, potentially explaining the cardiometabolic and autoimmune disorders [54], allergies [55,56], obesity [57], type 1 diabetes, increased body mass index (BMI) [57,58], liver problems and gastrointestinal disorders [55,59], and chronic immune disorders observed in children born via CS [55]. However, further studies are needed to eliminate confounding factors that may contribute to these outcomes. Another hypothesis involves the intrauterine exposure of the fetus to stress hormones, which are absent in the lack of natural stimuli occurring during vaginal delivery. The impact of maternal hormones, fetal hormone secretion, and the passage through the birth canal prepares the fetus for the transition to extrauterine life. In infants born via elective CS, this smooth transition is absent, suggesting that the initiation of labor before performing an elective CS could be beneficial [60]. Finally, the administration of antibiotics prior to CS and the use of synthetic oxytocin are still under investigation regarding their potential consequences on children’s health [55]. However, further studies are needed to eliminate confounding factors that may contribute to these outcomes.

Studies have also linked CS with behavioral disorders in children [61]. For instance, CS combined with low parental self-efficacy has been shown to predict a higher incidence of behavioral issues during the first months of life [62]. Additionally, CS has been associated with autism spectrum disorders (ASDs), with one proposed explanation being the influence of synthetic oxytocin. This hormone may impact behavior, mother–infant bonding, and even sexual behavior later in life. Children with autism spectrum disorders (ASDs) have been found to exhibit significantly lower plasma oxytocin levels compared to non-autistic children [63]. Furthermore, learning difficulties and attention-deficit/hyperactivity disorder (ADHD) [64] have also been associated with CS, presenting multiple hypotheses, such as the composition of gut microbiota, emergency CS, gestational age, and complications during delivery [65]. Additionally, it should be noted that maternal mental health directly impacts the psychosocial development of the child [61]. Maternal mental health issues affect the development of the mother–infant bond and breastfeeding practices [66,67] and therefore, it should be considered when assessing behavioral disorders in children. Table 2 provides a detailed overview of the immediate and long-term effects of CSs on neonates, infants, and children.

### 3.3. The Inappropriate and Low Utilization of CSs

On the other hand, in low-resource settings, the implications of CS are particularly significant due to constrained healthcare infrastructure, insufficient access to skilled professionals, and limited resources for adequate post-operative midwifery care [74]. The challenges often encountered pertain to adequate facilities, specialized personnel, availability of financial resources, and specialized care [75]. While global perinatal mortality remains a pressing public health concern, it is especially pronounced in Sub-Saharan Africa and Southeast Asia. These regions were responsible for 86% of the 295,000 maternal deaths in 2017 and 84% of the 2.5 million neonatal deaths and stillbirths that occur annually worldwide [76], since out of a total of 60% of global births, only 3.6% will deliver via CS [77]. For this reason, global attention has focused on the impact of healthcare quality on health outcomes, arguing that poor-quality midwifery and medical care, in addition to the lack of CSs, lead to a significant proportion of maternal and neonatal mortality and morbidity. Furthermore, economic inequalities within these countries significantly affect the mode of delivery, with limited access to emergency CS for the poorest subgroups and excessive use of CS without medical indication among wealthier subgroups [78]. However, some cultural factors may contribute to reduced use of health facilities, such as lack of privacy, shame, and the presence of male health professionals [79,80,81]. The factors influencing the reduced and inappropriate use of CS and their effect on maternal and child morbidity and mortality are presented in Table 3.

## 4. Discussion/Conclusions

This review aimed to investigate the effects of the global rapid increase in CS, as well as its inappropriate use, on maternal, neonatal, infant, and child health. The results indicated that CS is a contributing factor to maternal morbidity, which consequently increases the rates of long-term complications in subsequent pregnancies. Emergency CS also appears to have a more detrimental impact on maternal mental health and well-being. Immediate and long-term outcomes for the infant, in comparison to vaginal delivery, were also identified. Planned surgical deliveries appear to cause more immediate and long-term complications; however, further cohort studies with control for confounding factors are required, especially regarding the long-term consequences. An important long-term maternal complication that deserves further attention is isthmocele, also known as a cesarean scar defect. Isthmocele represents a pouch-like niche or dehiscence at the site of the uterine scar and is increasingly recognized as a consequence of repeated cesarean deliveries. Although often asymptomatic, it can lead to abnormal uterine bleeding, dysmenorrhea, chronic pelvic pain, dyspareunia, infertility, and complications in subsequent pregnancies, such as abnormal placentation and uterine rupture [82]. Furthermore, the COVID-19 pandemic has provided a unique temporal context for evaluating CS practices and outcomes. Studies conducted during this period reported altered obstetric management, with a tendency toward increased rates of elective or emergency CS due to infection control policies, fear of viral transmission, and limited staff availability. A retrospective study by Amadori et al. [83] demonstrated that peripartum outcomes during the lockdown period included changes in delivery timing, increased maternal anxiety, and variations in neonatal outcomes, reflecting the indirect effects of pandemic-related restrictions on obstetric care. These findings highlight the need to interpret CS outcomes within specific social and temporal contexts, as global crises such as COVID-19 may temporarily reshape delivery practices and influence maternal and neonatal health indicators.

On the other side, the low utilization of CS results in maternal and infant morbidity and mortality, reinforcing WHO recommendations for safe CS rates between 5 and 15% [1]. Therefore, policies and programs aimed at preventing unnecessary CS, while ensuring equitable access to facilities providing safe care, are essential.

The analysis of the results highlights that policies and practices regarding CS differ significantly between high- and low-resource countries. In low-income countries, limited access to safe facilities can increase maternal and neonatal morbidity and mortality, while in high-income countries, excessive use of cesarean section is associated with long-term complications. Strategies are needed that include educational programs for women and health professionals, midwife-assisted delivery rooms, and strict CS use protocols to promote the safe and appropriate use of cesarean sections, depending on the context. More specifically, we recommended (a) educational and support programs for women regarding the benefits of VD, as well as training for midwives and obstetricians; (b) creating structures that support the natural process of birth, such as midwife-assisted delivery rooms; (c) controlled use of CS with implementing protocols and guidelines to reduce unnecessary CS; (d) regularly assessing CS rates and developing policies to prevent their overuse; and (e) awareness and health education campaigns in developing countries, along with investment in access to quality maternal healthcare services.

This scoping review possesses several strengths that enhance the robustness and relevance of its findings. First, it provides a comprehensive synthesis of the most recent global evidence (2015–2025) on the short- and long-term effects of CS on maternal, neonatal, infant, and child health. Second, it systematically maps the literature according to the PRISMA-ScR guidelines, ensuring methodological transparency and reproducibility, and finally, the inclusion of studies from diverse geographical regions offers a wide perspective on both the overuse and underuse of cesarean sections across different healthcare systems and socioeconomic contexts. However, the review has certain limitations that should be acknowledged. First, despite the comprehensive search strategy applied across multiple databases (PubMed, Scopus, and Google Scholar), some relevant studies may have been missed, especially those published in languages other than English or in the gray literature sources. Second, the inclusion of heterogeneous study designs and outcome measures limited the ability to synthesize the results quantitatively. Therefore, the findings should be interpreted descriptively rather than as causal associations. Third, most of the included studies originated from high- and middle-income countries, which may restrict the generalizability of the findings to low-resource settings where CS utilization patterns and healthcare infrastructures differ significantly. Moreover, the included studies varied considerably in their design (retrospective, prospective, and cross-sectional), which may affect the comparability of the findings and limit the ability to draw causal inferences. This review did not include a formal quality assessment or risk-of-bias evaluation of the included studies, as this step is not mandatory within the PRISMA-ScR framework. Nonetheless, every effort was made to ensure methodological transparency and rigor throughout the review process. Finally, although systematic mapping of the literature provides a comprehensive overview, the absence of meta-analysis and formal quality assessment may introduce potential bias into the results. Therefore, the findings should be interpreted descriptively rather than causally.

## Figures and Tables

**Table 2:** Immediate and long-term effects of cesarean section on neonates, infants, and children.

Author/Year /Country	Sample	Design	Neonate Outcomes	Infant Outcomes	Child Outcomes
Black et al., 2015 [68], UK	321,287	Cohort study	-	-	Asthma and atopic sensitization
Kristensen & Henriksen, 2016 [59], Denmark	1,031,424	Cohort study	laryngitis, asthma, gastroenteritis	laryngitis, asthma, gastroenteritis	laryngitis, asthma, gastroenteritis
Papathoma et al., 2016 [56], Greece	233	Prospective study	Food allergies	Food allergies	Food allergies
O’Neill et al., 2016 [62], Sweden	125,766	Cohort study	-	-	Psychosis (after elective CS)
Liao et al., 2017 [69], Taiwan	579	Prospective study	-	Asthma, wheeze	-
Chiș et al., 2017 [70], Romania	72	Prospective study	Increased stress behaviors	-	-
Peters et al., 2018 [55], Australia	1545	Cohort study	Jaundice, feeding problems, hypothermia	Gastrointestinal disorders, eczema	Gastrointestinal disorders, increased odds of metabolic disorder, eczema
Bancalari et al., 2019 [71], Chile	324	Prospective study	Increased heart rate (2 h after elective CS)	-	-
Al-Zalabani et al., 2018 [63]	87	Case–control study	-	-	Autism spectrum disorders
Thomas et al., 2021 [51], Qatar	1466	Cohort study	Respiratory problems (mainly in neonates after elective CS)	-	-
Martín-Calvo et al., 2020 [57], Spain	599	Cohort study	-	-	Obesity and overweight
van Zadelhoff et al., 2023 [72], The Netherlands	314	Cohort study	Lower systolic blood pressure on first day of life (in neonates after elective CS)	-	-
Papadopoulou et al., 2023 [58], Greece	2946	Cohort study	-	-	Obesity and overweight
Chua et al., 2024 [54], Taiwan	675,718	Cohort study	-	-	Respiratory tract infections, asthma, allergic rhinitis, atopic dermatitis, obesity
Makri et al., 2024 [64], Greece	256	Case–control study	-	-	ADHD, specific learning disabilities

Notes: Neonate: from 0 to 28 days; infant: from 29 days to 1 year; child: from 1 year to 18 years [73]. Studies represent a wide range of geographic regions (Europe, Asia, and North and South America). Differences in study design (prospective vs. cohort vs. case–control) and period of data collection are indicated where available.

**Table 3:** The inappropriate use of cesarean section.

Author/Year /Country	Sample	Design	Exposure	Outcomes
Boatin et al., 2018 [78], US	72 low- and middle-income countries	Secondary analysis of demographic and health surveys	Economic inequalities	Limited access to emergency CS Excessive use of CS among wealthier subgroups
Chou et al., 2019 [81], US	81 low- and middle-income countries	Linear and deterministic model	Lack of healthcare infrastructures	Low quality of maternal and neonatal care
Dankwah et al., 2019 [77], Canada	4294 women from Ghana	Data from the 2014 Ghana Demographic and Health Survey	Inappropriate use of CS	CS below the WHO threshold for less wealthy societies CS above the WHO threshold for affluent classes
Ahmed et al., 2019 [79], Ethiopia	186 women from Ethiopia	Qualitative study	Cultural and social factors (preference for natural and fear of complications or doubts about safety of CSs)	Underutilisation of health services
Etcheverry et al., 2024 [80], France	504 women from 32 hospitals (8 per country)	Cross-sectional study	Shortage of specialized personnel, lack of privacy, and inadequate use of resources	CS overuse

## Supplementary Materials

The following supporting information can be downloaded at https://www.mdpi.com/article/10.3390/jcm14228102/s1. Table S1: PRISMA-ScR Checklist.

## Abbreviations

The following abbreviations are used in this manuscript:

CS	Cesarean Section
WHO	World Health Organization
